# Effect of Acidic Strength of Surface Ligands on the Carrier Relaxation Dynamics of Hybrid Perovskite Nanocrystals

**DOI:** 10.3390/nano13111718

**Published:** 2023-05-24

**Authors:** Sudhakar Narra, Po-Sen Liao, Sumit S. Bhosale, Eric Wei-Guang Diau

**Affiliations:** 1 Department of Applied Chemistry, National Yang Ming Chiao Tung University, Hsinchu 300093, Taiwan; sudhakar@nycu.edu.tw (S.N.); sam062986@gmail.com (P.-S.L.); sumitbhosale50@gmail.com (S.S.B.); 2 Center of Emergent Functional Matter Science, National Yang Ming Chiao Tung University, Hsinchu 300093, Taiwan

**Keywords:** perovskite nanocrystals, red emission, surface ligands, defect passivation, charge extraction, exciton, femtosecond TAS

## Abstract

Perovskite nanocrystals (PeNCs) are known for their use in numerous optoelectronic applications. Surface ligands are critical for passivating surface defects to enhance the charge transport and photoluminescence quantum yields of the PeNCs. Herein, we investigated the dual functional abilities of bulky cyclic organic ammonium cations as surface-passivating agents and charge scavengers to overcome the lability and insulating nature of conventional long-chain type oleyl amine and oleic acid ligands. Here, red-emitting hybrid PeNCs of the composition Cs_x_FA_(1−x)_PbBr_y_I_(3−y)_ are chosen as the standard (Std) sample, where cyclohexylammonium (CHA), phenylethylammonium (PEA) and (trifuluoromethyl)benzylamonium (TFB) cations were chosen as the bifunctional surface-passivating ligands. Photoluminescence decay dynamics showed that the chosen cyclic ligands could successfully eliminate the shallow defect-mediated decay process. Further, femtosecond transient absorption spectral (TAS) studies uncovered the rapidly decaying non-radiative pathways; i.e., charge extraction (trapping) by the surface ligands. The charge extraction rates of the bulky cyclic organic ammonium cations were shown to depend on their acid dissociation constant (pKa) values and actinic excitation energies. Excitation wavelength-dependent TAS studies indicate that the exciton trapping rate is slower than the carrier trapping rate of these surface ligands.

## 1. Introduction

Organic-inorganic halide perovskite nanocrystals (PeNCs) in the form ABX_3_ (A = organic/inorganic cations like Cesium cation (Cs^+^), methylammonium cations (MA), and formamidinium cations (FA); B = Pb^2+^ or Sn^2+^; X = Cl^−^, Br^−^ or I^−^) are now popularly being used in many applications such as LEDs, solar cells, photodetectors, photocatalysts, and so on [1,2,3,4,5,6,7,8]. The advantage of choosing these PeNCs over conventional semiconductors is their ease of preparation, impressive device performances, and the tunability of their excellent optoelectronic properties [3,9]. The competitive device performances of PeNCs are attributed to the low defect density and high activation energies required for the formation of deep defects. The major hurdles for commercializing PeNCs are their long-term stability, ion migration, hysteresis, and degradation caused by oxygen and water vapor [10,11,12,13,14].

The surface chemistry of the encapsulating ligands plays a prominent role in modulating the optical properties and stabilities of the PeNCs [3,8,13,15,16,17,18,19,20,21,22,23,24]. Conventionally, PeNCs synthesized by the hot injection/addition methods utilize oleylamine (OLA) and oleic acid (OA) as encapsulating ligands [1,25]. The labile nature of these ligands leads to the loss of surface ligands during purification [25], affecting the stability, whereas the insulation nature of these ligands inhibits efficient charge conduction [23]. Several surface engineering strategies have been worked out to passivate the surface defects and improve the charge conduction properties of the PeNCs [15,18,19,24,26,27,28,29,30,31,32]. Among them, utilizing cyclic aromatic cations to simultaneously passivate the surfaces and induce interfacial energy transfer is an interesting approach to boost the conduction properties of the PeNCs [15,26,27]. The acid–base chemistry between the uncoordinated lead ions and the encapsulating surface ligands is of importance, as the former, being a soft acid, prefers a soft base to achieve efficient passivation of the surface [17,31]. Despite efficient passivation by soft base-type ligands, the photoluminescence quantum yields in most of the cases are less than unity [19]. The mechanisms of the non-radiative losses induced by passivating ligands need deeper understanding.

In this study, we show the passivating effects of the bulky cyclic/aromatic cations (short-chain ligands) on synthesized red-emitting Cs_x_FA_(1−x)_PbBr_y_I_(3−y)_ (CsFA) PeNCs. The short-chain ligands used to passivate the surfaces of CsFA PeNCs are cyclohexylammonium (CHA), phenylethylammonium (PEA) and 4-(trifluoromethyl)benzylamonium (TFB). Photoluminescence decay studies show efficient passivation of shallow trap states by these short-chain ligands. Further, femtosecond transient absorption spectral (TAS) experiments have been performed to uncover the nonradiative losses in the PeNCs. It is shown that surface ligands are responsible for the ultrafast nonradiative losses induced in these PeNCS, and the rates of trapping are shown to correlate with the pKa values of the surface ligands.

## 2. Materials and Methods

### 2.1. Chemicals

Lead iodide (PbI_2_) (purity 99%, Sigma Aldrich, St Louis, MO, USA), Cesium carbonate (Cs_2_CO_3_) (purity 99%, Thermo Fisher Scientific, Germany), Formamidinium bromide (FABr) (purity 99%, Greatcell Solar Materials, Queanbeyan, NSW, Australia), Phenylethylammonium iodide (PEAI) (purity 99%, Greatcell Solar Materials, Queanbeyan, NSW, Australia), Cyclohexylmethylammonium iodide (CHAI) (purity 99%, Greatcell Solar Materials, Queanbeyan, NSW, Australia), 4-Trifluoromethyl-benzylammonium iodide (TFBI) (purity 99%, Greatcell Solar Materials, Queanbeyan, NSW, Australia), Oleylamine (OLA) (Technical grade, purity 70%, Sigma Aldrich, Zwijndrecht, The Netherlands), Oleic acid (OA) (Technical grade, purity 90%, Sigma Aldrich, Zwijndrecht, The Netherlands), Octadecene (Technical grade, purity 90%, Sigma Aldrich, St. Louis, MO, USA), Hydriodic acid (57% in water, Alfa Aesar, Kandel, Germany), Isopropanol (IPA) (anhydrous, purity 99.5%, Sigma Aldrich, Steinheim am Albuch, Germany), Methyl acetate (Purity 99%, Thermo Fisher Scientific, Germany), Octane (Purity 99.8%, J T Baker Chemicals B V., Deventer, The Netherlands).

### 2.2. Synthesis of Perovskite Nanocrystals

The PeNCs used in this work were synthesized using the one-pot sequential hot addition method (HAM) reported elsewhere [33]. CsFA PeNCs were prepared from the precursor solutions of Cs_2_CO_3_, FABr, and PbI_2_, whereas surface passivation of CsFA PeNCs were performed by adding the precursor solutions of the bulky organic cations. A volume of 0.94 mmol of Cs_2_CO_3_ precursor solution was prepared by adding 3 mL of OA and 3 mL of octadecane to a 20 mL vial, and the resultant mixture was heated at 150 °C until the solution became transparent. A volume of 1.88 mmol FABr solution was prepared by dissolving precursor salt in 3 mL IPA. Then, 1.88 mmol of PbI_2_ in octadecane was taken in a 50 mL round-bottomed (RB) flask and stirred for one hour at 100 °C to remove water. Later, 5 mL each of OA and OLA were added to dissolve the PbI_2_. The PbI_2_ solution changes from turbid to clear after complete dissolution of PbI_2_. The temperature of the PbI_2_ solution was further raised to 140 °C and the FABr solution was added. The reaction mixture turned a turbid orange color in the RB flask. The temperature was slowly increased, and the solution turned to light yellow color from turbid orange color during this process. When the temperature became 200 °C, 6 mL of cesium oleate was added to the reaction mixture, and then the RB flask carrying the reaction mixture was immediately immersed in an ice bath to arrest the further growth of the PeNCs to the black phase. This completed the synthesis of FACs PeNCs. The surface-passivated PeNCs’ synthesis involves an additional step, wherein 1.88 mmol of the corresponding bulky organic ammonium iodides (CHAI, PEAI, and TFBI) in IPA were added to the reaction mixture together with the FABr before the injection of cesium oleate. The synthesized PeNCs were purified using repetitive washing and centrifugation methods as described. Crude PeNC solution was centrifuged at 9000 rpm for 40 min. The precipitate containing large particles was discarded. The supernatant is the desired PeNC sample of the desired size. The surface-passivated PeNCs need further purification. Methyl acetate is added at a ratio of 1:3 to precipitate PeNCs. The precipitates collected by centrifugation were finally dispersed in octane to obtain the final products. The synthesized PeNCs are reported to display a relative photoluminescence quantum yield of 0.72, 0.76, 0.70, and 0.69, respectively for Std, CHA, PEA, and TFB samples [33]. The pKa values of the short-chain ligands were obtained by measuring the pH of the corresponding 0.1 M aqueous salt solutions. 

### 2.3. Optical Characterizations

UV-Visible absorption spectra of PeNCs samples were measured using a JASCO V-780 absorption spectrophotometer equipped with a JASCO integration sphere accessory. The absorption spectra of PeNC samples were measured with a custom cell holder equipped with BK9 glass windows with an optical pathlength of 0.2 mm. The absorption spectra of the samples were corrected for reflection and scattering effects. The absorbance of the PeNC samples was estimated using Equation (1) as shown below.

(1) $$A=-\log(\frac{I_{T}}{\left(I_{0}-I_{R}\right)})$$

where *I*_0_ and *I_T_* are the transmittances of the reference light and samples, respectively, and *I_R_* is the reflectance of the sample.

The PL spectra of the PeNC samples were measured using a customized PL spectrometer. The PL spectrometer is equipped with a 150 W Xe lamp from OBB as a light source. The excitation wavelength was chosen using a Horiba microHR spectrometer (1200 groves grating, blaze wavelength: 330 nm). The emission from the sample was collected using a TRIAX spectrometer (600 groves grating, blaze wavelength: 500 nm). The collected emission was focused on the exist slit of the monochromator, collected by the photomultiplier tube connected to the preamplifier. The signal was digitized using a SpecACQ2 digitizer. All the electronic components are controlled by software in a coordinated way to obtain the PL spectrum. The excitation wavelength was set to 450 nm for measuring the PL spectrum of the samples. The excitation light scattering was minimized by using a 480 nm long pass color glass filter on the emission side of the spectrometer.

The PL lifetime measurements of PeNC samples were performed using a PicoQuant FluoTime 200 fluorescence lifetime spectrometer. The PeNCs were excited using a 375 nm picosecond diode laser pulse (LDH-P-C -375, FWHM: 60 ps), controlled by a diode laser driver PLD 200-B. The emission from the sample was collected by a double monochromator and detected using a high-speed microchannel plate photomultiplier tube (MCP-PMT). The MCP-PMT output was controlled and digitized by a PicoHarp 300 controller. The PL lifetimes of PeNCs were measured in the range of 620–640 nm. 

The fs-transient absorption spectra were acquired using an Excipro ultrafast pump-probe absorption spectrometer (CDP systems). The TAS spectrometer was coupled with a femtosecond Ti: Sapphire amplified laser system (Coherent Legend USP, 795 nm, 1 kHz, 3 mJ, 35 fs), optical parametric amplifiers (TOPAS-C), and a sapphire plate for generating femtosecond pump and probe pulses. The details of the TAS system are described elsewhere [34]. Briefly, 480 nm and 640 nm pump pulses generated from TOPAS-C are used for the above bandgap and the near-resonant pumping of PeNC samples dispersed in an octane solution. The probe pulses are generated by focusing the weaker portion of the fundamental beam on a sapphire plate to generate a white light continuum, which is limited to 750 nm using a short pass filter. The pump-induced changes in the absorption of the sample are measured by optically modulating the pump pulses using an optical chopper. The fluctuation in the probe light spectrum is nullified by referencing the probe light. The time-dependent absorption changes in the sample are obtained by delaying the pump pulses with respect to the probe pulse. The polarization between pump and probe pulses is set to a magic angle to suppress the coherent artifacts. The transient absorption spectra are corrected for the white light dispersion using the optical Kerr signals of the solvent [35], and denoised using singular value decomposition (SVD) analysis. 

## 3. Results

**Figure 1:**
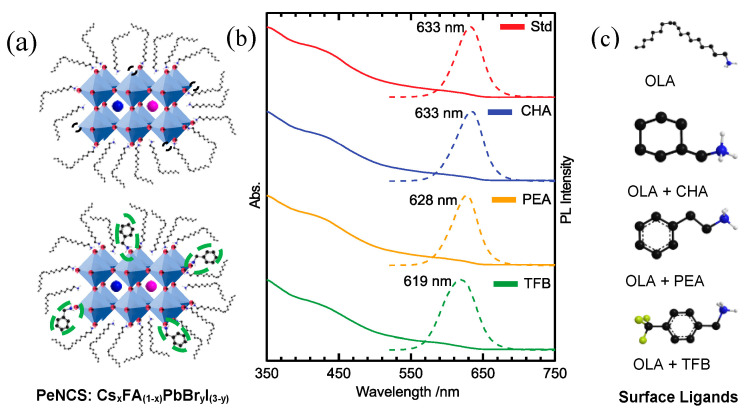
(**a**) Schematic of Cs_x_FA_(1−x)_PbBr_y_I_(3−y)_ PeNCs showing halogen vacancies and their passivation by short-chain ligands, (**b**) UV-PL spectra of Std, CHA, PEA and TFB PeNC samples and (**c**) chemical structures of surface-passivating short-chain ligands.

The schematic of CsFA PeNCs (Std) with surface-passivating long-chain ligand OLA and halogen vacancies is shown in Figure 1a. The halogen vacancies on the surface were passivated using the short-chain ligands CHA, PEA, and TFB as shown in Figure 1a,c, respectively. Further, the surface-passivating effects of short-chain ligands CHA, PEA, TFB on the Std PeNCs were examined using the optical spectroscopic techniques described below. The surface-passivated PeNCs hereafter are referred to using their ligand abbreviation names.

### 3.1. UV-PL Spectroscopy

UV-Vis absorption and PL spectra of PeNCs are shown in Figure 1b. The absorption spectra of nanocrystal samples show the onset of absorption at 620 nm, while their emission peaks were centered at 626 ± 7 nm, indicating that the synthesized nanocrystals are red-emitting nanocrystals suitable for red LED applications with a narrow bandwidth of 20 nm. The modest shifts in PL peak positions between the samples might be due to the modulation of the composition proportions. It is worth mentioning that all optical measurements are performed from diluted samples/thinner path length cell holders to maintain similar optical densities for comparison purposes, and also to minimize the reabsorption effects on the PL spectra. These precautions were taken to carefully evaluate the passivating ligands’ effects on the optical properties of the samples.

Further, the UV-Vis absorption spectra of the PeNC samples were fitted using Elliot’s equations (Figure S1) described elsewhere [36,37,38]. The bandgaps estimated from Elliot’s fit (Table S1) correlate well with the corresponding sample’s PL emission maximum, whereas the estimated exciton-binding energies (Table S1) are extremely small, implying excitons can split to free carriers easily, which is a common phenomenon for large-sized crystals [38,39].

### 3.2. TCSPC Decay Kinetics

**Figure 2:**
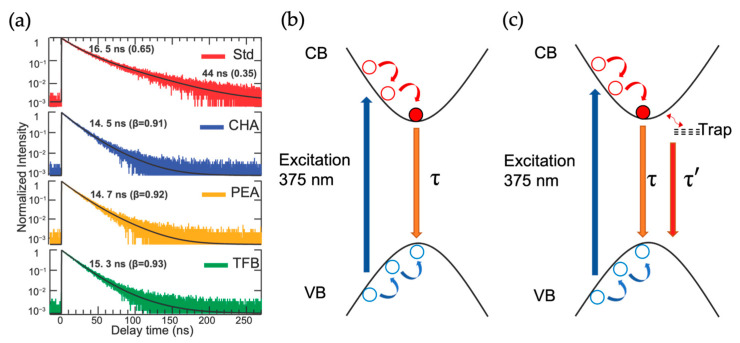
(**a**) Overlay of TCSPC decay profiles and curve fits of Std, CHA, PEA, and TFB PeNCs. The decay profiles were monitored at their respective PL maxima positions of the samples. (**b**,**c**) PL decay mechanism for the surface-passivated and standard PeNC samples, respectively.

The TCSPC decay profiles of PeNC samples are shown in Figure 2. The decay profiles shown in Figure 2 were collected from respective PL maxima. The TCSPC decay profiles of PeNCs show an instantaneous rise and a complete decay within 400 ns. The transient decay profiles were fitted using either exponential or stretched exponential functions. The Std PeNCs samples fit well to the bi-exponential decaying function described by Equation (2), while the CHA, PEA, and TFB samples were fitted to a stretched exponential function described by Equation (3).

(2) $$I\left(t\right)=A_{1}\exp\left(-\frac{t}{\tau_{1}}\right)+A_{2}\exp\left(-\frac{t}{\tau_{2}}\right)$$

(3) $$I\left(t\right)=A\;{\exp\left(-\frac{t}{\tau }\right)}^{\beta }$$

Here, $A_{1}$, $A_{2}$, and A are preexponential factors, $\tau_{1}$, $\tau_{2}$, and τ are time coefficients, and β (which lies between 0 and 1) is the stretching exponent that expresses the degree of asymmetry of the synthesized PeNCs.

The Std PeNC decays with time constants of 16.5 ns and 44 ns, as shown in Figure 2, whereas surface-passivated samples decay with a time constant of 15 ns and β close to unity (mono-exponential behavior), indicating that the samples are either homogenous in nature or the emission centers of the PeNCs are of similar origin. Wavelength-dependent decay profiles (Figure S2) display longer lifetimes on the longer wavelength side of their respective emission spectra, which is indicative of inter-crystal energy transfer in the PeNCs due to exciton hopping [39,40]. Further, the observed 16.5 ns and 44 ns decaying time constants for the Std PeNC sample were attributed to direct and shallow trap state-mediated recombination, as described by Figure 2c. The assignments of the faster decaying components to direct recombination and slower recombination with delayed luminescence caused by shallow trap states has also been observed for other types of PeNC samples [41,42]. The effective surface-passivating nature of the bulky organic ligands is clearly described by their TCSPC decay profiles, as the shallow trap state-mediated recombination is completely quenched, as shown in the relaxation mechanism in Figure 2b. However, the TCSPC results presented in this section are limited to the sub-nanosecond and nanosecond decay dynamics, the nonradiative processes occurring in the sub-picosecond time scales, and the reasons for the non-unity PLQY are scant in these studies. Hence, fs TAS experiments were performed to uncover the faster dynamics of the PeNC samples.

### 3.3. TAS Decay Dynamics: Above Bandgap Excitation

**Figure 3:**
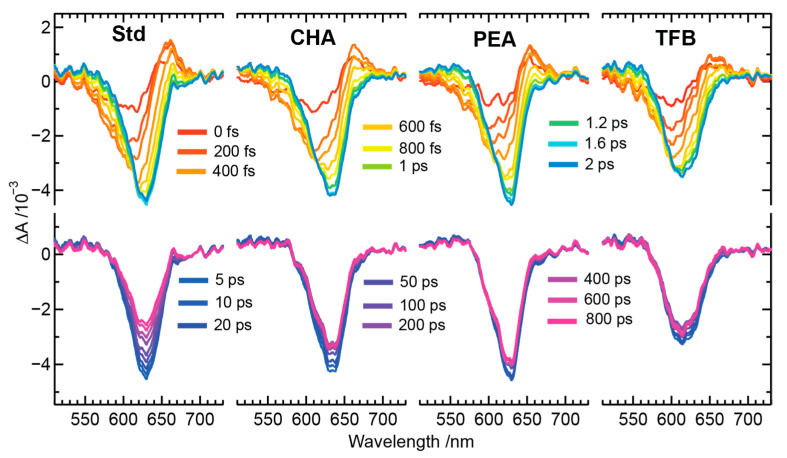
TAS profiles of Std, CHA, PEA, and TFB PeNC samples of the composition Cs_x_FA_(1−x)_PbBr_y_I_(3−y)_. The actinic excitation wavelength for the samples was set to 480 nm.

The TAS profiles of PeNC samples for the above bandgap excitation condition were performed by exciting the samples using 480 nm actinic pump pules, and probing between 530 and 730 nm using broadband white-light continuum pulses, as shown in Figure 3. The top panels show the evolution of the TAS profiles acquired between 0–2 ps of pump-probe delay times, whereas the bottom panels display the subsequent recombination of the TAS profiles acquired between 5–800 ps of pump-probe delay times. The TAS profile shapes of all PeNC samples resemble each other, except for the differences in their bleach minima positions and their decay dynamics. Further, the TAS profiles shown between 0–2 ps reflect the signatures of hot carrier distributions, as described earlier [43,44], whereas recombination decays at later stages can be seen in the TAS profiles shown between 5–800 ps. The decay-assisted decomposition of TAS profiles was deconvoluted using the modeling of the kinetic profiles using a relaxation scheme which will be discussed later.

### 3.4. TAS Decay Dynamics: Near-Resonant Excitation

**Figure 4:**
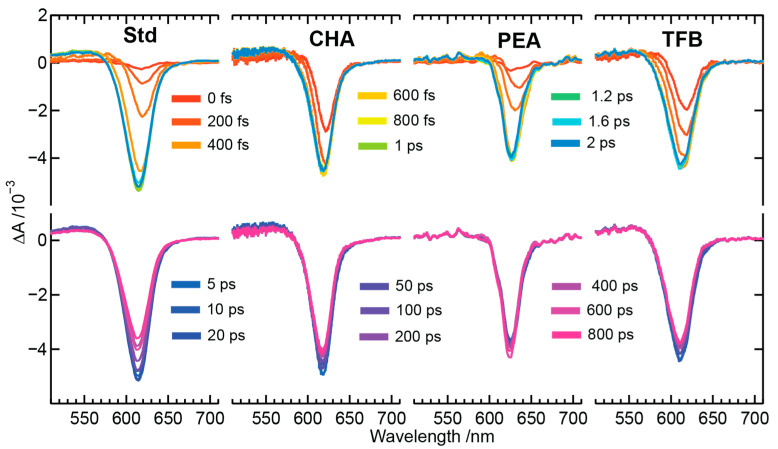
TAS profiles of perovskite nanocrystals of the composition Cs_x_FA_(1−x)_PbBr_y_I_(3−y)_ (Std), and surface-passivated perovskite crystals with bulkier organic ligands referred as CHA, PEA, and TFB in the display panel. The excitation wavelength of the perovskite nanocrystals was set to 640 nm.

The TAS profiles of PeNC samples in the near-resonant excitation condition was performed by exciting the samples using 640 nm actinic excitation pulses, while the probing region is set similar to the above band gap excitation, as shown in Figure 4. Near-resonant excitation experiments were performed to eliminate the hot carrier profiles from the TAS profiles and focus only on the recombination dynamics of the excitons or cold carriers. Similar to the above band gap excitation, the top panels show the evolution of the TAS profiles acquired between 0–2 ps of pump–probe delay times, whereas the bottom panels display the subsequent recombination of the TAS profiles acquired between 5–800 ps of pump–probe delay times. The TAS profile shapes of all PeNC samples acquired under near-resonant conditions are sharper and smoother than the above bandgap excitations. The sharper band shapes confirm the absence of hot carriers under near-resonant conditions, whereas smoother band shapes could be due to weaker scattering from the PeNCs under 640 nm excitation. Further, the TAS profiles shown between 0–2 ps display no photoinduced absorption (PIA) band on the blue side of the PB band, unlike the above bandgap excitation. The PIA band on the blue side of the PB band is assigned to the population of forbidden excitonic state due to the breaking of inversion symmetry for PeNCs [45]. The decay-assisted decomposition of TAS profiles was deconvoluted using the modeling of the kinetic profiles, using a relaxation scheme which will be discussed later.

### 3.5. Decay-Assisted Spectral Deconvolution: Kinetic Modeling of TAS Profiles

**Figure 5:**
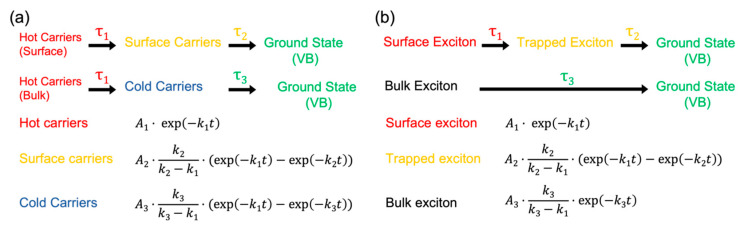
Carrier (**a**) and exciton (**b**) relaxation kinetic models for PeNCs under 480 nm (above band gap) and 640 nm (near-resonance) excitation conditions, respectively.

The TAS profile spectrograms were subjected to singular-value decomposition (SVD) analysis for both the above bandgap and near-resonant excitation conditions to find the minimum resolvable spectral features in both of the experiments. SVD analysis suggests that at least three components (Figure S3) are required to model the relaxation kinetics based on their decay dynamics. Based on spectral features and energies of excitation, a free carrier model is applied for above band gap excitation, whereas the exciton model is applied for near-resonant excitation, as shown in Figure 5. The choice of the models can be justified as the above bandgap excitation energy is sufficient to split the weakly binding excitons to hot carriers, and similarly, resonant excitation with the excitonic transitions should deplete exciton bands, yielding more excitons, as evidenced by the sharp PB bands for the near-resonant excitation condition.

**Figure 6:**
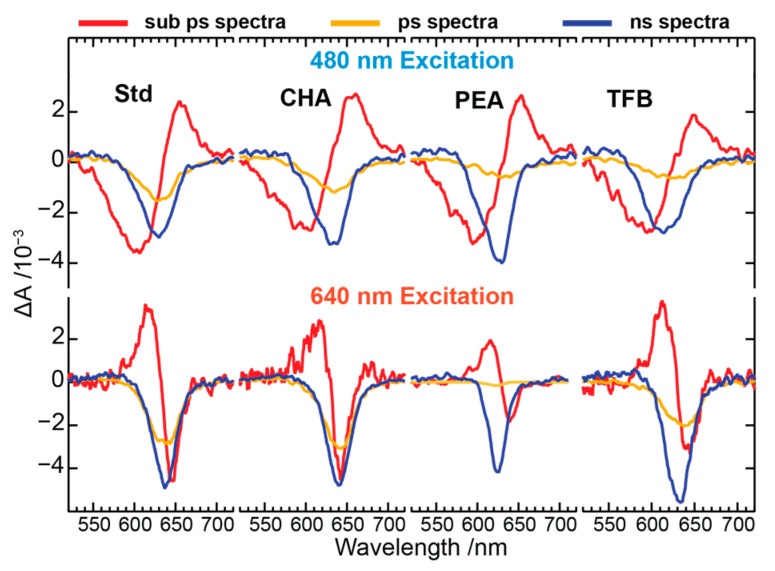
Decay-assisted deconvoluted spectral profiles of Std, CHA, PEA, and TFB PeNCs of the composition Cs_x_FA_(1−x)_PbBr_y_I_(3−y)_ (Std).

The kinetic models described in Figure 5 are sequential parallel models describing the heterogeneous carrier/exciton relaxation processes, namely in the bulk and in the proximity of surface defects. Photoexcitation with actinic excitation pulses produces an excess hot carriers/excitons depending on the excitation energy, which progressively cools down and leads to recombination of cold carriers in bulk PeNC, whereas the hot carriers/excitons when produced close to the proximity of surface defect will be captured by the defect sites, which are referred to as surface carriers/excitons. They recombine non-radiatively via an alternative pathway to that of bulk PeNC. The wavelength-dependent TAS profiles shown in Figures S4–S11 were fitted to their respective kinetic models to deconvolute the decay-assisted spectra, as shown in Figure 6. The kinetic models are validated by global fitting of the TAS kinetic profiles at all probe wavelengths. Further residual maps shown in Figures S4–S11 were checked to see the authenticity of the data fitting according to the kinetic model (Figure 5). The featureless residual maps confirm the validity of our fitting approach. The fitting coefficients obtained from the curve fitting of TAS profiles using the kinetic models are shown in Table 1 and Table 2.

The hot carrier/exciton cooling times are shown to be independent of the surface ligand passivation effects, as shown in Table 1 and Table 2, with a cooling time of 0.5 and 0.1 ps, respectively, for the hot carriers and excitons. However, surface carrier/exciton recombination time coefficients showed similar changes with changes in the composition of the surface ligands, as shown in Table 1 and Table 2. The surface carriers/exciton recombination seem to correlate with the pK_a_ values of the surface ligands. The estimated pK_a_ values of the surface ligands decrease in the order of PEA (12.14) > CHA (11.68) > TFB (10.89). The surface carrier/exciton recombination also follows the same order as the pKa, with pristine samples being slowest due to the insulating nature of the OA/OLA ligands. The smaller the pK_a_ the higher the affinity to accept the electrons.

## 4. Discussion

**Figure 7:**
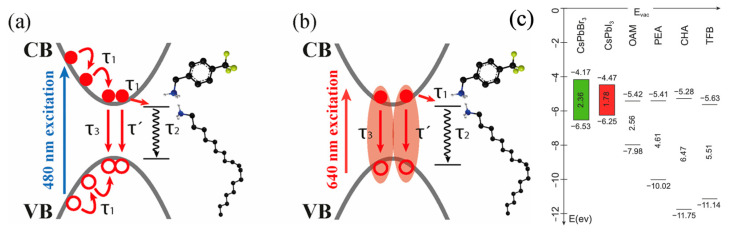
Relaxation mechanism of Cs_x_FA_(1−x)_PbBr_y_I_(3−y)_ perovskite nanocrystals under (**a**) 480 nm and (**b**) 640 nm excitation conditions. (**c**) HOMO-LUMO estimations of the bulkier cyclic aromatic cations using DFT method. The VB and CB band positions of CsPbBr_3_ and CsPbI_3_ were taken from the literature [47].

The PL lifetimes of PeNCs showed both radiative and delayed luminescence processes on the nanosecond timescale, whereas TAS results demonstrate a non-radiative channel with a timescale of under 100 ps. The delayed luminescence process occurring in the nanosecond timescales are often attributed to shallow defects stemming from iodine vacancies, whereas the picosecond non-radiative processes shown in this work could be due to the charge or exciton transfer/trapping to the surface-passivating ligand, as shown elsewhere [41,46]. The short-chain surface ligands successfully blocked the shallow trap-mediated recombination; however, they opened up another charge/exciton funneling channel. So, DFT calculations (basis: B3LYP/631 + g(d, p), charge: singlet) were performed to estimate the HOMO and LUMO levels of the surface ligands to check the energetic alignments with that of pristine CsPbBr_3_ and CsPbI_3_ nanocrystals, as shown in Figure 7c. The LUMO levels of the ligands lie deeper in the forbidden gap of PeNCs, and therefore the picosecond non-radiative recombination process observed in the TAS experiments could be that of charge/exciton trapping to the surface ligand, whose rates correlate well with the pKa values of the ligands. Further, the relaxation model showing the charge and exciton trapping under the above band gap and resonant excitations are shown in Figure 7a and Figure 7b, respectively. The above bandgap produces hot carriers that cool within about ~0.5 ps (τ_1_), and surface ligand-mediated recombination occurs within 100 ps (τ_2_), following Std (108 ps) > PEA (45 ps) > CHA (42 ps) > TFB (22 ps), whereas band-to-band (τ_3_) and shallow trap-mediated recombination (τ^’^, only for Std sample) occur on nanosecond timescales, as described in the TCSPC decay section. Similarly, resonant excitation produces hot excitons that are rapidly trapped by surface ligands within 0.1 ps (τ_1_), and the surface ligand-trapped excitons (τ_2_) recombine slightly slower than the trapped carriers as follows: Std (168 ps) > PEA (100 ps) > CHA (92 ps) > TFB(49 ps). However, band-to-band (τ_3_) and shallow trap-mediated recombination (τ^’^, only for Std sample) occur in nanosecond times scales. Further, it is interesting to note that pKa is the dominating effect, rather than the aromaticity, as CHA and PEA samples display similar charge-trapping rates due to their closer pKa values.

The PL decay and TAS results presented in this study show both the passivation and charge-scavenging nature of the surface ligands. The charge separation/scavenging caused by the short-chain ligands may be beneficial for photovoltaic or photocatalytic applications in which charges have to be extracted, whereas in the scenarios of retaining the radiative recombination, surface ligands with weaker acidity are preferred. A surface ligand with weak acidity is also less prone to deprotonation, which is beneficial for the long-term stability of the PeNCs.

## 5. Conclusions

Red-emitting PeNCs of the composition Cs_x_FA_(1−x)_PbBr_y_I_(3−y)_ were shown to have emission at 626 ± 7 nm, with small exciton binding energies. The short-chain ligands CHA, PEA, and TFB cations used in this study are shown to exert weak modulation on the electronic bandgap. The TCSPC decay profiles of the Std PeNCs were shown to decay with two decaying time coefficients of 16.5 ns (direction recombination) and 44 ns (shallow-trap mediated recombination), respectively, while short-capping ligand-passivated samples decay via a stretched exponential function with uniform time coefficients of about 15 ns, and the stretch factor *β* is close to 0.9. The absence of long-lived decay components in the CHA, PEA, and TFB samples confirms the passivation effects of the short-chain ligands. Femtosecond TAS studies of the nanocrystals using 480 nm excitation show the spectral signatures of hot carries, surface carriers, and cold carriers. The hot carriers recombine with 0.5 ps for all samples, and cold carrier recombination was not complete, while the surface carrier recombination rates show variation in the order Std (108 ps) > PEA (46 ps) > CHA (42 ps) > TFB (22 ps). The increased reaction rates of surface carriers can be explained based on the pKa, as the pKa shows the order PEA (12.14) > CHA (11.68) > TFB (10.89). The decrease in pKa is associated with the increased electron-accepting ability of the ligands. The charge losses at the surface of ligands were ascribed to the non-unity PLQY of these red-emitting nanocrystals. Femtosecond TAS studies of the PeNCs under resonant excitation showed the spectral signatures of the bulk exciton, surface exciton, and trapped exciton. It is shown that surface exciton becomes trapped in 0.1 ps in all samples, and bulk excitation is not complete in all the samples, while trapped excitons recombine non-radiatively in a similar manner to surface carriers, but with varied decay coefficients: Std (168 ps) > PEA (100 ps) > CHA (92 ps) > TFB (49 ps). The trapped excitons recombine slower when compared to surface carriers due to the variation in the interactions between surface ligands and carriers or excitons.

## Figures and Tables

**Table 1:** Carrier model fitting coefficients obtained from the global fitting of TAS profiles obtained using 480 nm actinic excitation pulses. τ_1_ is the hot carrier cooling time, τ_2_ is surface carrier recombination time and τ_3_ is the ns offset.

Sample	τ_1_ (ps)	τ_2_ (ps)
Std.	0.5	108
CHA	0.6	42
PEA	0.5	45
TFB	0.5	22

**Table 2:** Exciton model fitting coefficients obtained from the global fitting of TAS profiles obtained using 640 nm actinic excitation pulses. τ1 is the hot carrier cooling time, τ2 is surface exciton recombination time and τ3 is the ns offset.

Sample	τ_1_ (ps)	τ_2_ (ps)
Std.	0.1	168
CHA	0.1	92
PEA	0.2	100
TFB	0.1	49

## Data Availability

Data can be obtained from the corresponding author upon a reasonable request.

## Supplementary Materials

The following supporting information can be downloaded at: https://www.mdpi.com/article/10.3390/nano13111718/s1, Figure S1: UV-Vis absorption spectra of (a) Std, (b) CHA, (c) PEA and (d) TFB PeNC samples of the composition Cs_x_FA_(1−x)_PbBr_y_I_(3−y)_ (Std). The band edges of the absorption spectra were fitted to Elliot’s model equations, which is expressed as a linear combination of excitonic and continuum states. The contributions of excitonic, continuum and the total fits of the band edges are overlaid on the absorption profiles.; Figure S2: Wavelength dependent TCSPC decay profiles (a) Std, (b) CHA, (c) PEA and (d) TFB PeNC samples of the composition Cs_x_FA_(1−x)_PbBr_y_I_(3−y)_ (Std). The Std sample decay profiles were fitted to a bi-exponential function whereas surface passivated PeNC samples decay profiles were fitted to a strectched exponential function.; Figure S3: Singular value plots (a, e) Std, (b, f) CHA, (c, g) PEA and (d, h) TFB PeNC samples of the composition Cs_x_FA_(1−x)_PbBr_y_I_(3−y)_ (Std). The top and bottom panels results represent 480 and 640 nm excitation conditions. These plots suggests that at least three species are involved in the relaxation dynamics of perovskite nanocrystals irrespective of excitation and surface passivation conditions.; Figure S4: Wavelength dependent transient absorption decay kinetic profiles of (a) Cs_x_FA_(1−x)_PbBr_y_I_(3−y)_ (Std) PeNCs. The decay kinetics were fitted with a kinetic model shown in Figure 5a. The TA data were obtained using 480 nm excitation condition. Spectrogram of Std (b), reconstructed spectrogram (c) obtained from the global curve fitting analysis and residual spectrogram (d) obtained by subtracting b from c. The featureless residual spectrogram serves as a validation of the kinetic model.; Figure S5: Wavelength dependant transient absorption decay kinetic profiles of (a) Cs_x_FA_(1−x)_PbBr_y_I_(3−y)_ PeNCs passivated with short chain ligand CHA. The decay kinetics were fitted with a kinetic model shown in Figure 5a. The TA data were obtained using 480 nm excitation condition. Spectrogram of CHA (b), reconstructed spectrogram (c) obtained from the global curve fitting analysis and residual spectrogram (d) obtained by subtracting b from c. The featureless residual spectrogram serves as a validation of the kinetic model.; Figure S6: Wavelength dependent transient absorption decay kinetic profiles of (a) Cs_x_FA_(1−x)_PbBr_y_I_(3−y)_ PeNCs passivated with short chain ligand PEA. The decay kinetics were fitted with a kinetic model shown in Figure 5a. The TA data were obtained using 480 nm excitation condition. Spectrogram of PEA (b), reconstructed spectrogram (c) obtained from the global curve fitting analysis and residual spectrogram (d) obtained by subtracting b from c. The featureless residual spectrogram serves as a validation of the kinetic model.; Figure S7: Wavelength dependent transient absorption decay kinetic profiles of (a) Cs_x_FA_(1−x)_PbBr_y_I_(3−y)_ PeNCs passivated with short chain ligand TFB. The decay kinetics were fitted with a kinetic model shown in Figure 5a. The TA data were obtained using 480 nm excitation condition. Spectrogram of TFB (b), reconstructed spectrogram (c) obtained from the global curve fitting analysis and residual spectrogram (d) obtained by subtracting b from c. The featureless residual spectrogram serves as a validation of the kinetic model.; Figure S8: Wavelength dependent transient absorption decay kinetic profiles of (a) Cs_x_FA_(1−x)_PbBr_y_I_(3−y)_ (Std) PeNCs. The decay kinetics were fitted with a kinetic model shown in Figure 5b. The TA data were obtained using 640 nm excitation condition. Spectrogram of Std (b), reconstructed spectrogram (c) obtained from the global curve fitting analysis and residual spectrogram (d) obtained by subtracting b from c. The featureless residual spectrogram serves as a validation of the kinetic model.; Figure S9: Wavelength dependent transient absorption decay kinetic profiles of (a) Cs_x_FA_(1−x)_PbBr_y_I_(3−y)_ (Std) PeNCs passivated with short chain ligand CHA. The decay kinetics were fitted with a kinetic model shown in Figure 5b. The TA data were obtained using 640 nm excitation condition. Spectrogram of CHA (b), reconstructed spectrogram (c) obtained from the global curve fitting analysis and residual spectrogram (d) obtained by subtracting b from c. The featureless residual spectrogram serves as a validation of the kinetic model.; Figure S10: Wavelength dependent transient absorption decay kinetic profiles of (a) Cs_x_FA_(1−x)_PbBr_y_I_(3−y)_ (Std) PeNCs passivated with short chain ligand PEA. The decay kinetics were fitted with a kinetic model shown in Figure 5b. The TA data were obtained using 640 nm excitation condition. Spectrogram of PEA (b), reconstructed spectrogram (c) obtained from the global curve fitting analysis and residual spectrogram (d) obtained by subtracting b from c. The featureless residual spectrogram serves as a validation of the kinetic model. Figure S11: Wavelength dependent transient absorption decay kinetic profiles of (a) Cs_x_FA_(1−x)_PbBr_y_I_(3−y)_ (Std) PeNCs passivated with short chain ligand TFB. The decay kinetics were fitted with a kinetic model shown in Figure 5b. The TA data were obtained using 640 nm excitation condition. Spectrogram of TFB (b), reconstructed spectrogram (c) obtained from the global curve fitting analysis and residual spectrogram (d) obtained by subtracting b from c. The featureless residual spectrogram serves as a validation of the kinetic model. Figure S12: Femtosecond transient absorption spectrograms of (a) Std, (b) CHA, (c) PEA and (d) TFB PeNC samples of the composition Cs_x_FA_(1−x)_PbBr_y_I_(3−y)_ (Std). The spectrograms were obtained using 480 nm excitation pulses. Figure S13: Femtosecond transient absorption spectrograms of ((a) Std, (b) CHA, (c) PEA and (d) TFB PeNC samples of the composition Cs_x_FA_(1−x)_PbBr_y_I_(3−y)_ (Std). The spectrograms were obtained using 640 nm excitation pulses; Table S1: Estimations of bandgaps and exciton binding energies obtained from UV-Vis absorption spectral band edge fitting using Elliot model equation for Cs_x_FA_(1−x)_PbBr_y_I_(3−y)_ (Std) and surface passivated perovskite nanocrystals with bulky organic ligands CHA, PEA, and TFB.
