# Supplementary material for: Effect of Acidic Strength of Surface Ligands on the Carrier Relaxation Dynamics of Hybrid Perovskite Nanocrystals
Source: Nanomaterials (Basel). 2023 May 24;13(11):1718. doi: 10.3390/nano13111718 (PMC10254759; doi:10.3390/nano13111718)
Supplement: Supplementary file 1 [file nanomaterials-13-01718-s001.zip › nanomaterials-2399558-supplementary.pdf]

Effect of Acidic Strength of Surface Ligands on the Carrier Relaxation Dynamics of Hybrid Perovskite Nanocrystals

Sudhakar Narra[1,2], Po-Sen Liao[1], Sumit S. Bhosale[1] and Eric Wei-Guang Diau[1,2,*]

[1] Department of Applied Chemistry, National Yang Ming Chiao Tung University, Hsinchu 300093, Taiwan.
[2] Center of Emergent Functional Matter Science, National Yang Ming Chiao Tung University, Hsinchu 300093, Taiwan
* Correspondence: diau@nycu.edu.tw (E.W.-G.D.)

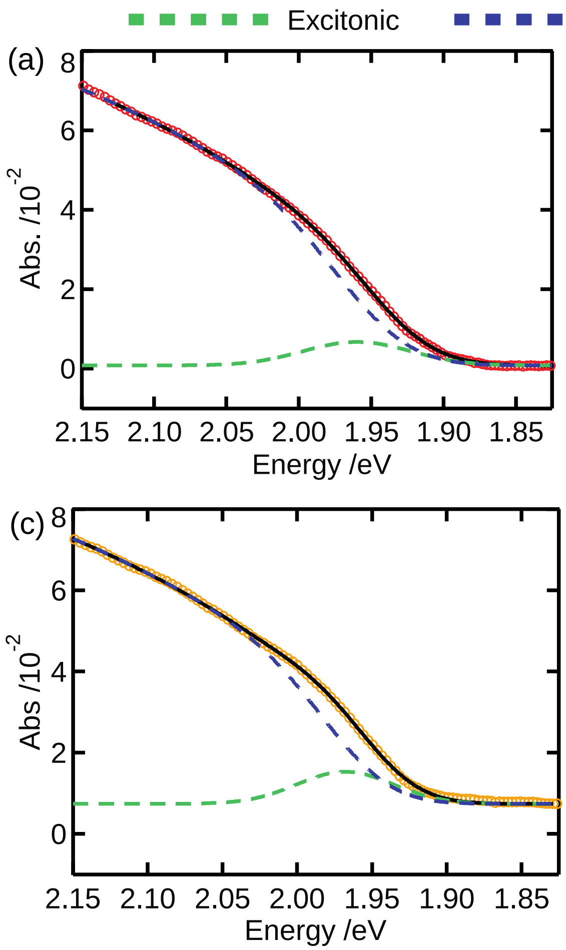

Figure S1. UV-Vis absorption spectra of (a) Std, (b) CHA, (c) PEA and (d) TFB PeNC samples of the composition $Cs_xFA_{(1-x)}PbBr_yI_{(3-y)}$ (Std). The band edges of the absorption spectra were fitted to Elliot's model equation, which is expressed as a linear combination of excitonic and continuum states. The contributions of excitonic, continumm and the total fits of the band edges are overlaid on the absorption profiles.

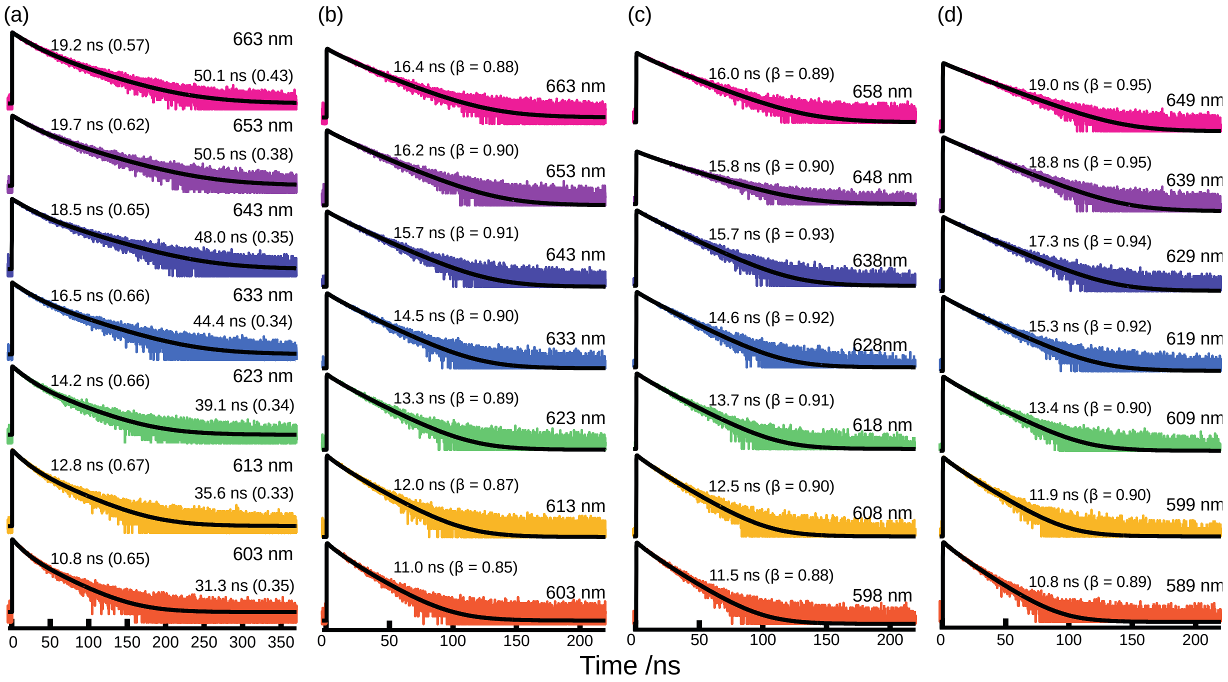

Figure S2. Wavelength dependent TCSPC decay profiles (a) Std, (b) CHA, (c) PEA and (d) TFB PeNC samples of the composition $Cs_xFA_{(1-x)}PbBr_yI_{(3-y)}$ (Std). The Std sample decay profiles were fitted to a bi-exponential function whereas surface passivated PeNC samples decay profiles were fitted to a strectched exponential function.

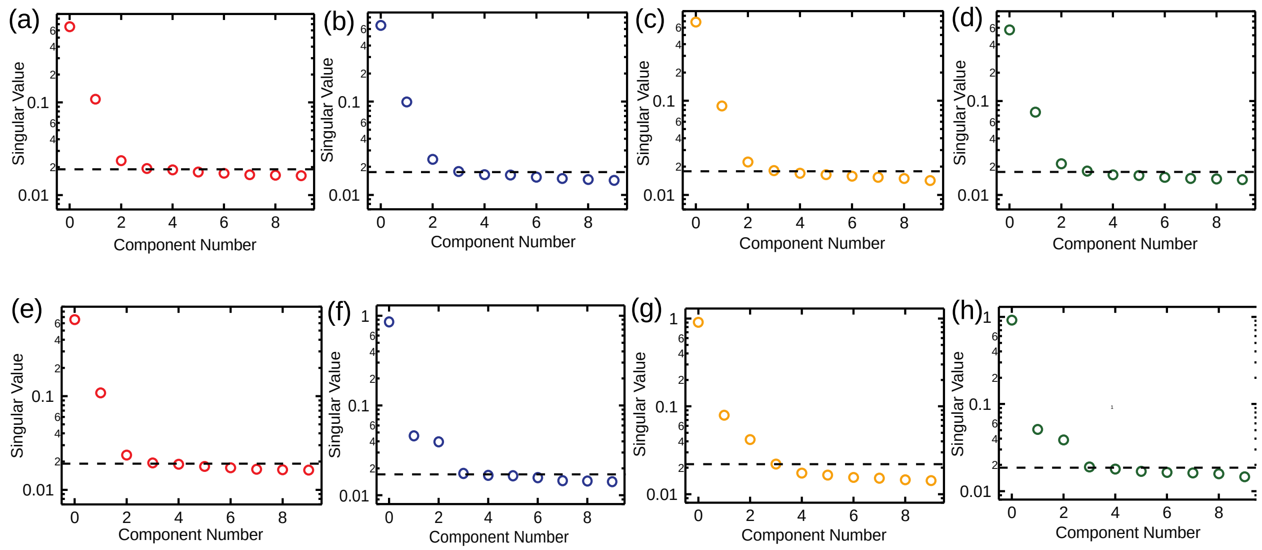

Figure S3. Singular value plots (a, e) Std, (b, f) CHA, (c, g) PEA and (d, h) TFB PeNC samples of the composition $Cs_xFA_{(1-x)}PbBr_yI_{(3-y)}$ (Std). The top and bottom panels results represent 480 and 640 nm excitation conditions. These plots suggest that at least three species are involved in the relaxation dyanmics of perovskite nanocrystals irrespective of excitation and surface passivation conditions.

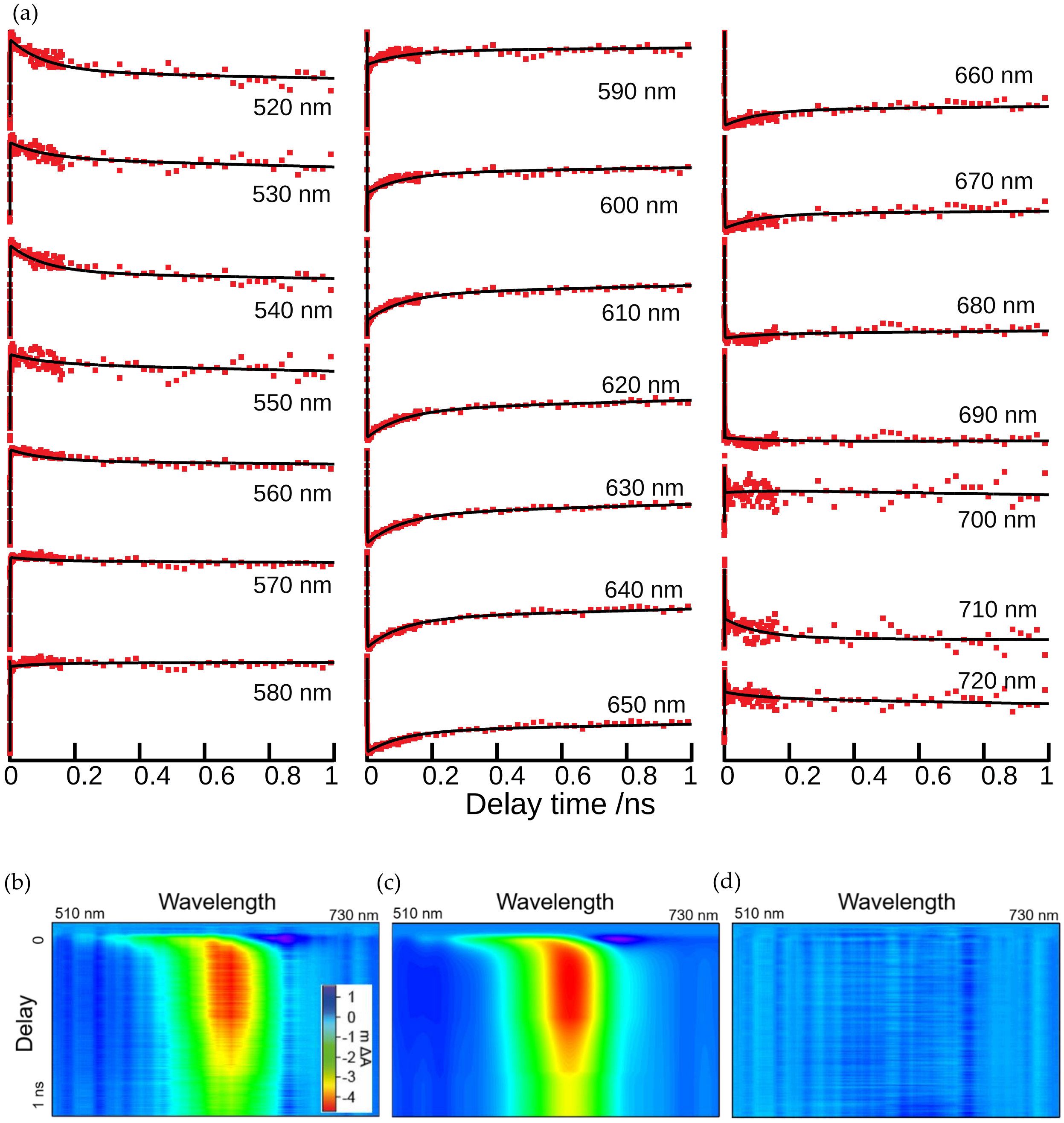

Figure S4. Wavelength dependent transient absorption decay kinetic profiles of (a) $Cs_xFA_{(1-x)}PbBr_yI_{(3-y)}$ (Std) PeNCs. The decay kinetics were fitted with a kinetic model shown in Figure 5a. The TA data were obtained using 480 nm excitation condition. Spectrogram of Std (b), reconstructed spectrogram (c) obtained from the global curve fitting analysis and resuidual spectrogram (d) obtained by substracting b from c. The featureless residual spectrogram serves as a validation of the kinetic model.

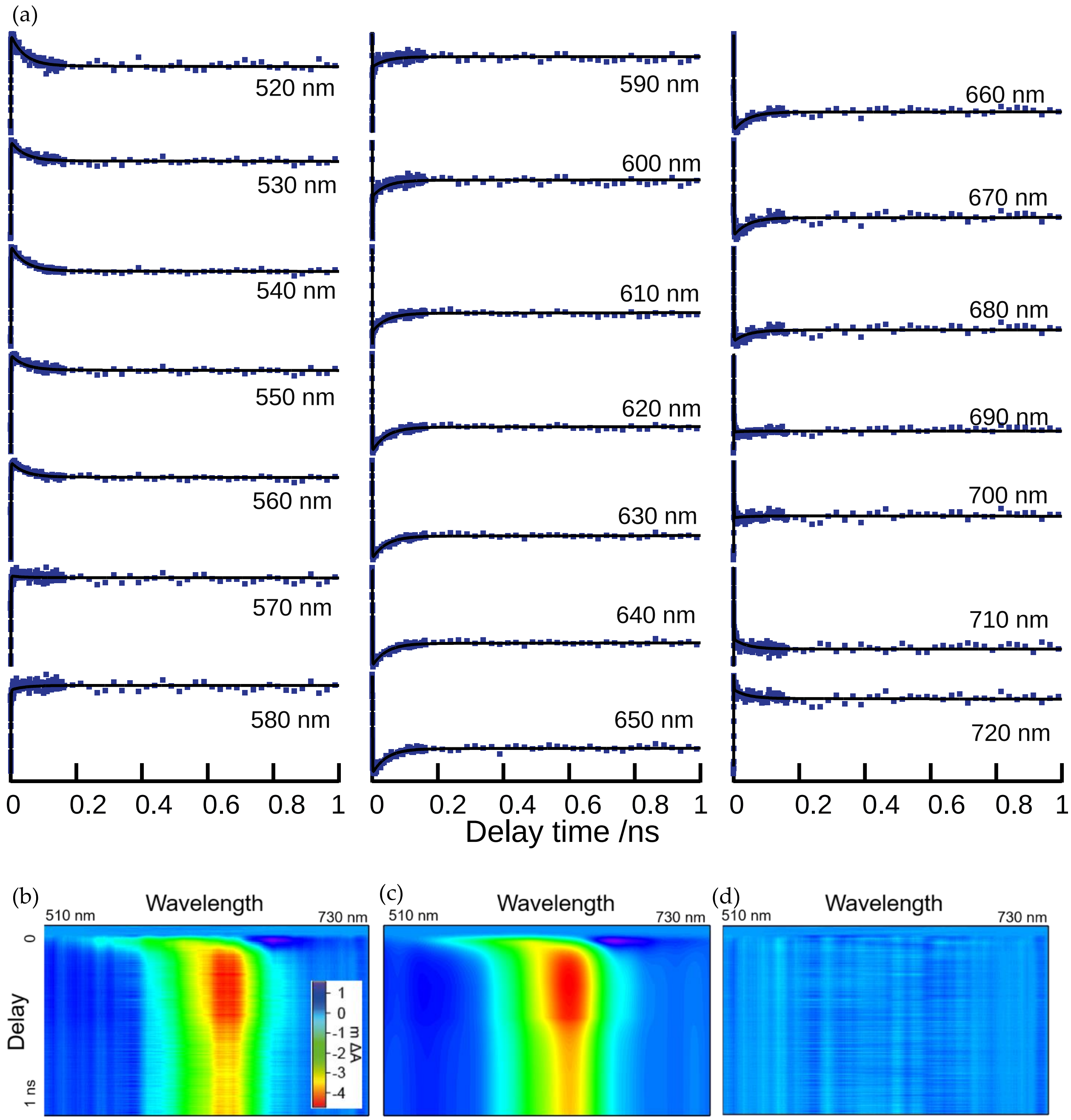

Figure S5. Wavelength dependent transient absorption decay kinetic profiles of (a) $Cs_xFA_{(1-x)}PbBr_yI_{(3-y)}$ PeNCs passivated with short chain ligand CHA. The decay kinetics were fitted with a kinetic model shown in Figure 5a. The TA data were obtained using 480 nm excitation condition. Spectrogram of CHA (b), reconstructed spectrogram (c) obtained from the global curve fitting analysis and residual spectrogram (d) obtained by substracting b from c. The featureless residual spectrogram serves as a validation of the kinetic model.

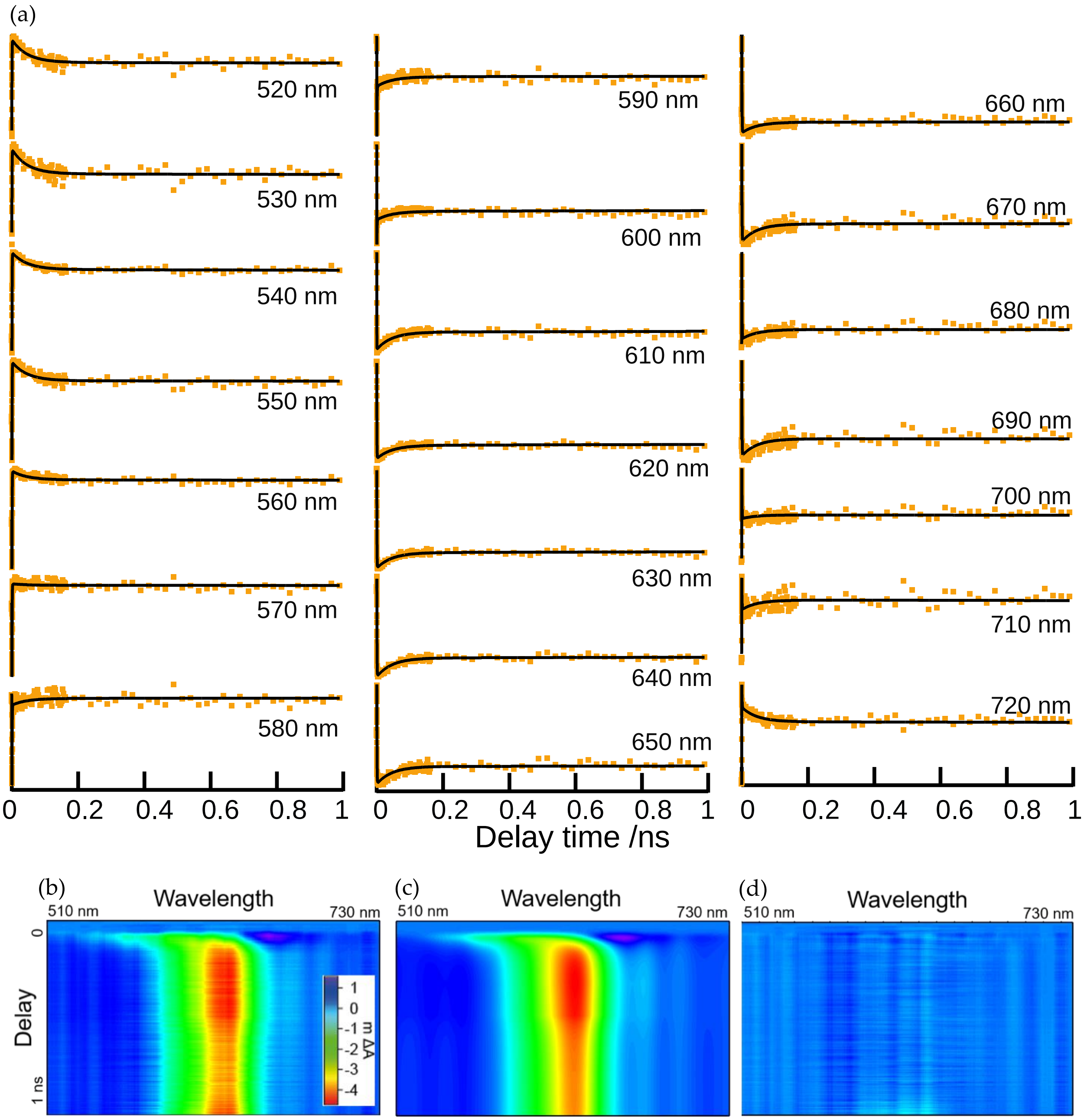

Figure S6. Wavelength dependent transient absorption decay kinetic profiles of (a) $Cs_xFA_{(1-x)}PbBr_yI_{(3-y)}$ PeNCs passivated with short chain ligand PEA. The decay kinetics were fitted with a kinetic model shown in Figure 5a. The TA data were obtained using 480 nm excitation condition. Spectrogram of PEA (b), reconstructed spectrogram (c) obtained from the global curve fitting analysis and residual spectrogram (d) obtained by substracting b from c. The featureless residual spectrogram serves as a validation of the kinetic model.

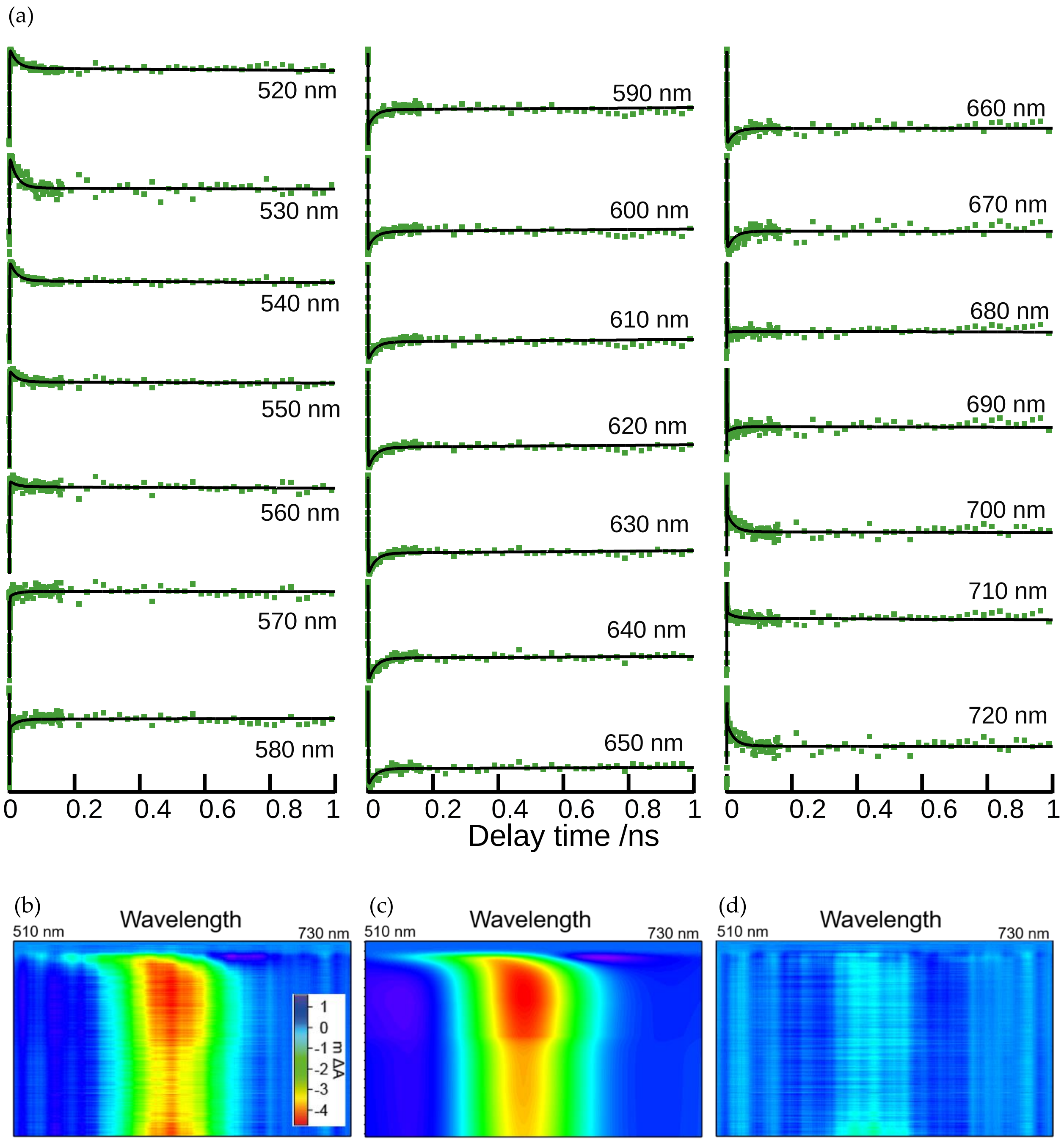

Figure S7. Wavelength dependent transient absorption decay kinetic profiles of (a) $Cs_xFA_{(1-x)}PbBr_yI_{(3-y)}$ PeNCs passivated with short chain ligand TFB. The decay kinetics were fitted with a kinetic model shown in Figure 5a. The TA data were obtained using 480 nm excitation condition. Spectrogram of TFB (b), reconstructed spectrogram (c) obtained from the global curve fitting analysis and residual spectrogram (d) obtained by substracting b from c. The featureless residual spectrogram serves as a validation of the kinetic model.

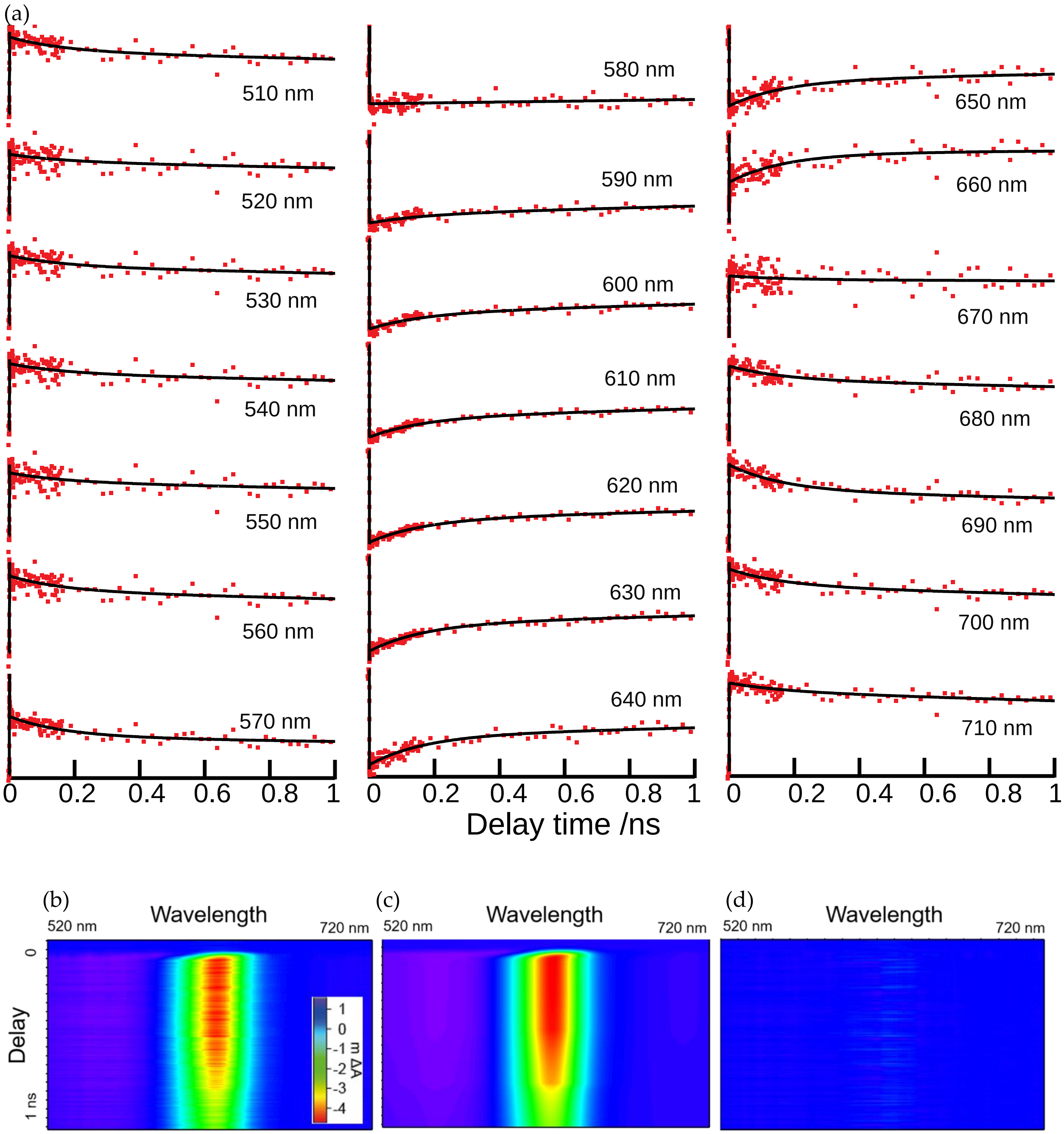

Figure S8. Wavelength dependent transient absorption decay kinetic profiles of (a) $Cs_xFA_{(1-x)}PbBr_yI_{(3-y)}$ (Std) PeNCs. The decay kinetics were fitted with a kinetic model shown in Figure 5b. The TA data were obtained using 640 nm excitation condition. Spectrogram of Std (b), reconstructed spectrogram (c) obtained from the global curve fitting analysis and residual spectrogram (d) obtained by substracting b from c. The featureless residual spectrogram serves as a validation of the kinetic model.

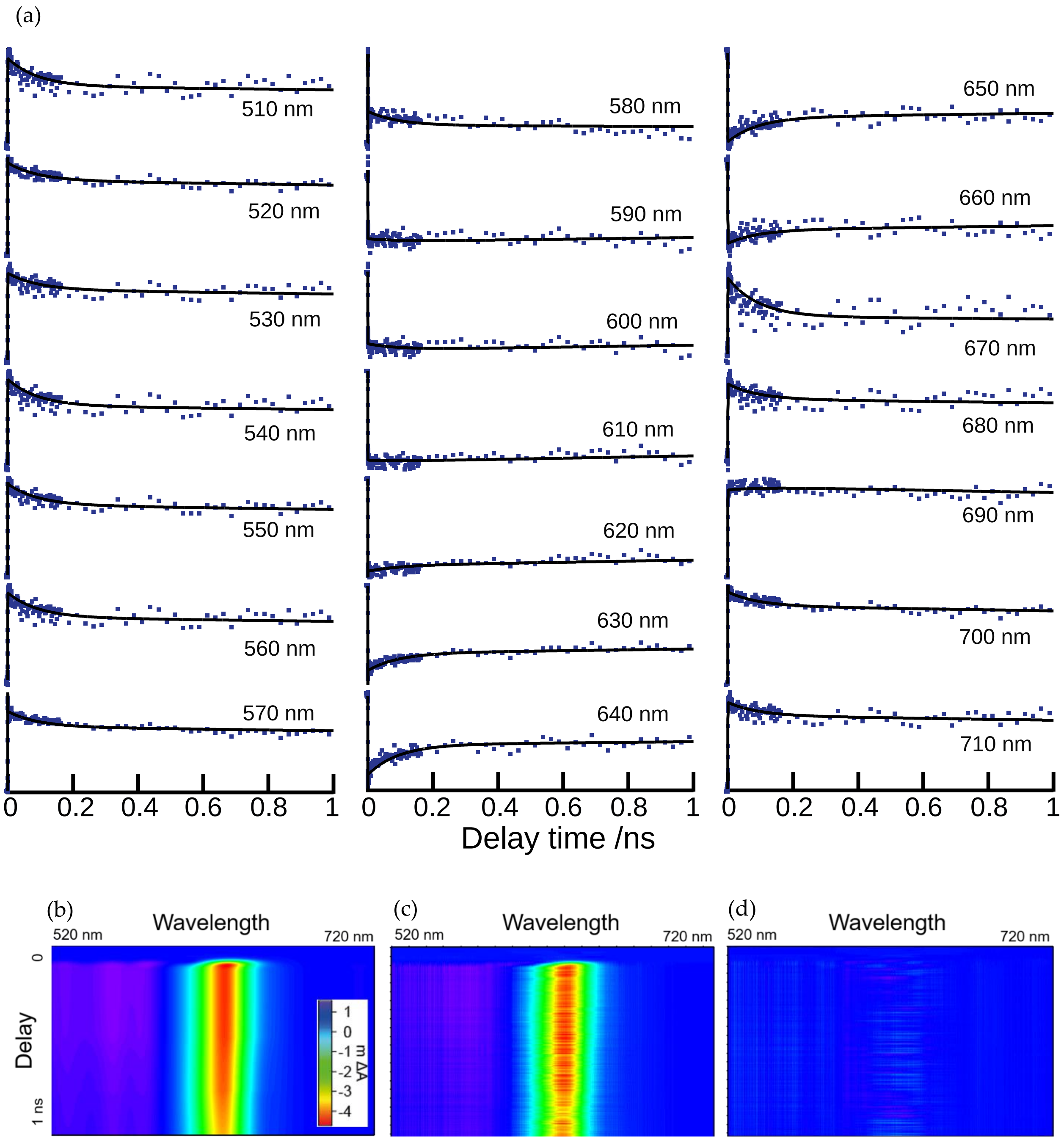

Figure S9. Wavelength dependent transient absorption decay kinetic profiles of (a) $Cs_xFA_{(1-x)}PbBr_yI_{(3-y)}$ (Std) PeNCs passivated with short chain ligand CHA. The decay kinetics were fitted with a kinetic model shown in Figure 5b. The TA data were obtained using 640 nm excitation condition. Spectrogram of CHA (b), reconstructed spectrogram (c) obtained from the global curve fitting analysis and residual spectrogram (d) obtained by substracting b from c. The featureless residual spectrogram serves as a validation of the kinetic model.

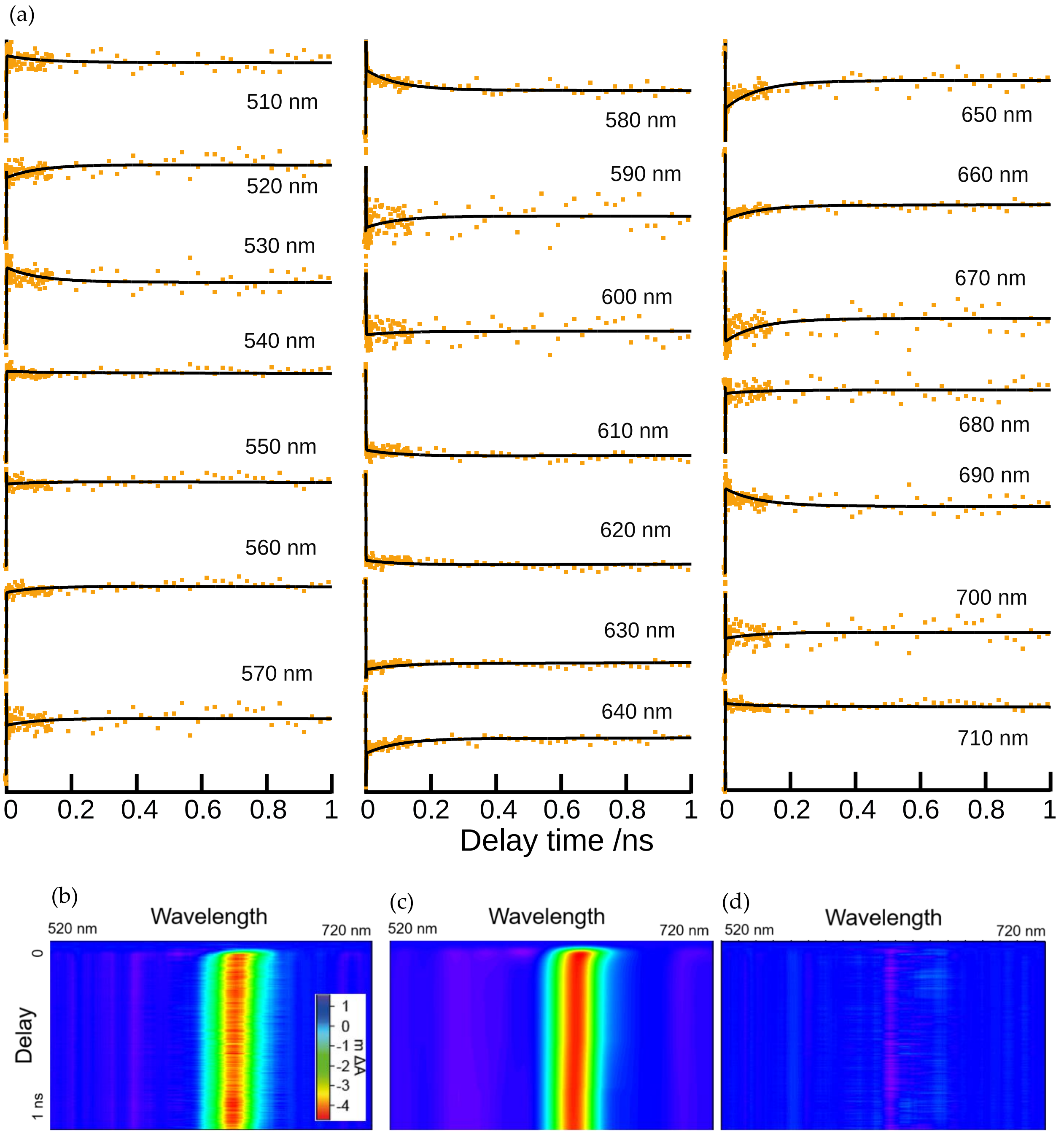

Figure S10. Wavelength dependent transient absorption decay kinetic profiles of (a) $Cs_xFA_{(1-x)}PbBr_yI_{(3-y)}$ (Std) PeNCs passivated with short chain ligand PEA. The decay kinetics were fitted with a kinetic model shown in Figure 5b. The TA data were obtained using 640 nm excitation condition. Spectrogram of PEA (b), reconstructed spectrogram (c) obtained from the global curve fitting analysis and residual spectrogram (d) obtained by substracting b from c. The featureless residual spectrogram serves as a validation of the kinetic model.

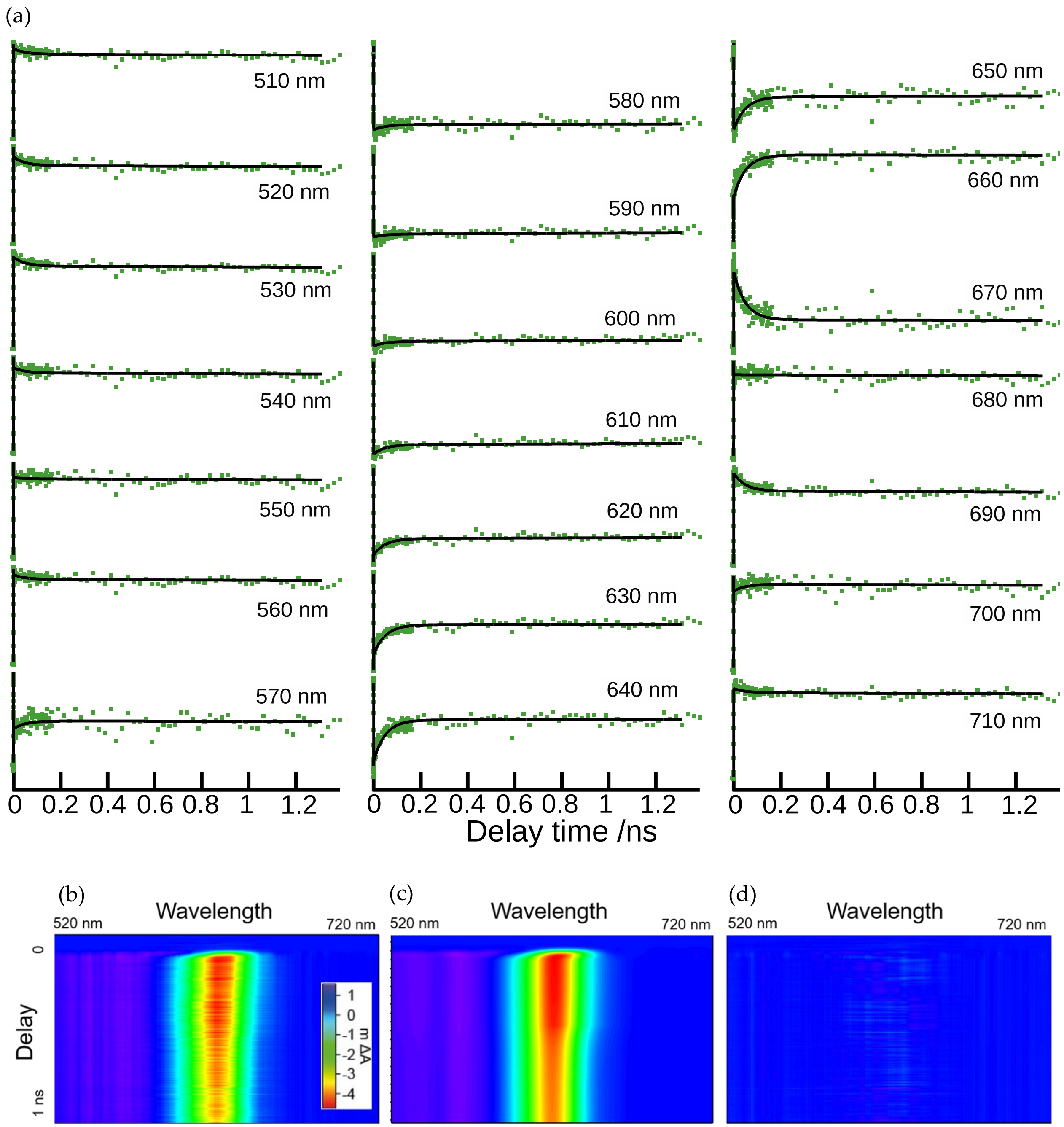

Figure S11. Wavelength dependent transient absorption decay kinetic profiles of (a) $Cs_xFA_{(1-x)}PbBr_yI_{(3-y)}$ (Std) PeNCs passivated with short chain ligand TFB. The decay kinetics were fitted with a kinetic model shown in Figure 5b. The TA data were obtained using 640 nm excitation condition. Spectrogram of TFB (b), reconstructed spectrogram (c) obtained from the global curve fitting analysis and residual spectrogram (d) obtained by substracting b from c. The featureless residual spectrogram serves as a validation of the kinetic model.

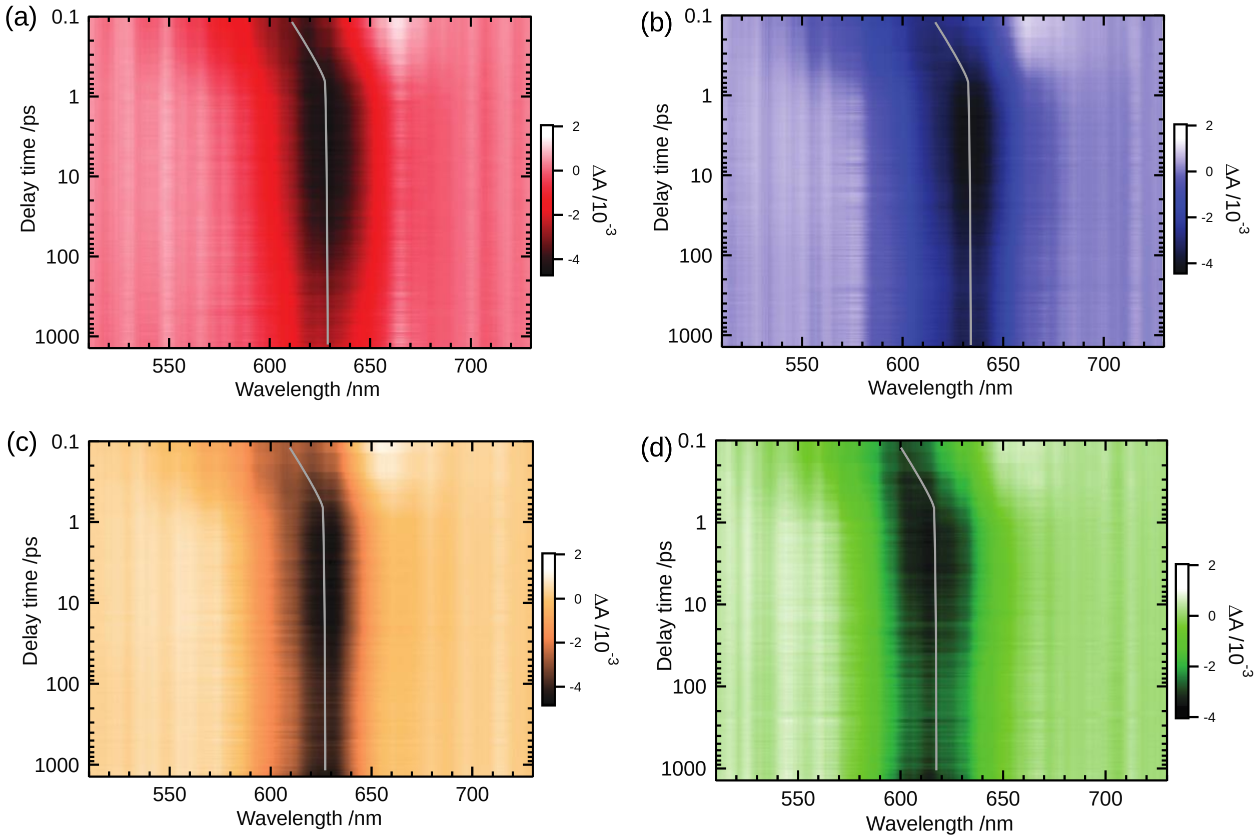

Figure S12. Femtosecond transient absorption spectrograms of (a) Std, (b) CHA, (c) PEA and (d) TFB PeNC samples of the composition $Cs_xFA_{(1-x)}PbBr_yI_{(3-y)}$ (Std). The spectrograms were obtained using 480 nm excitation pulses.

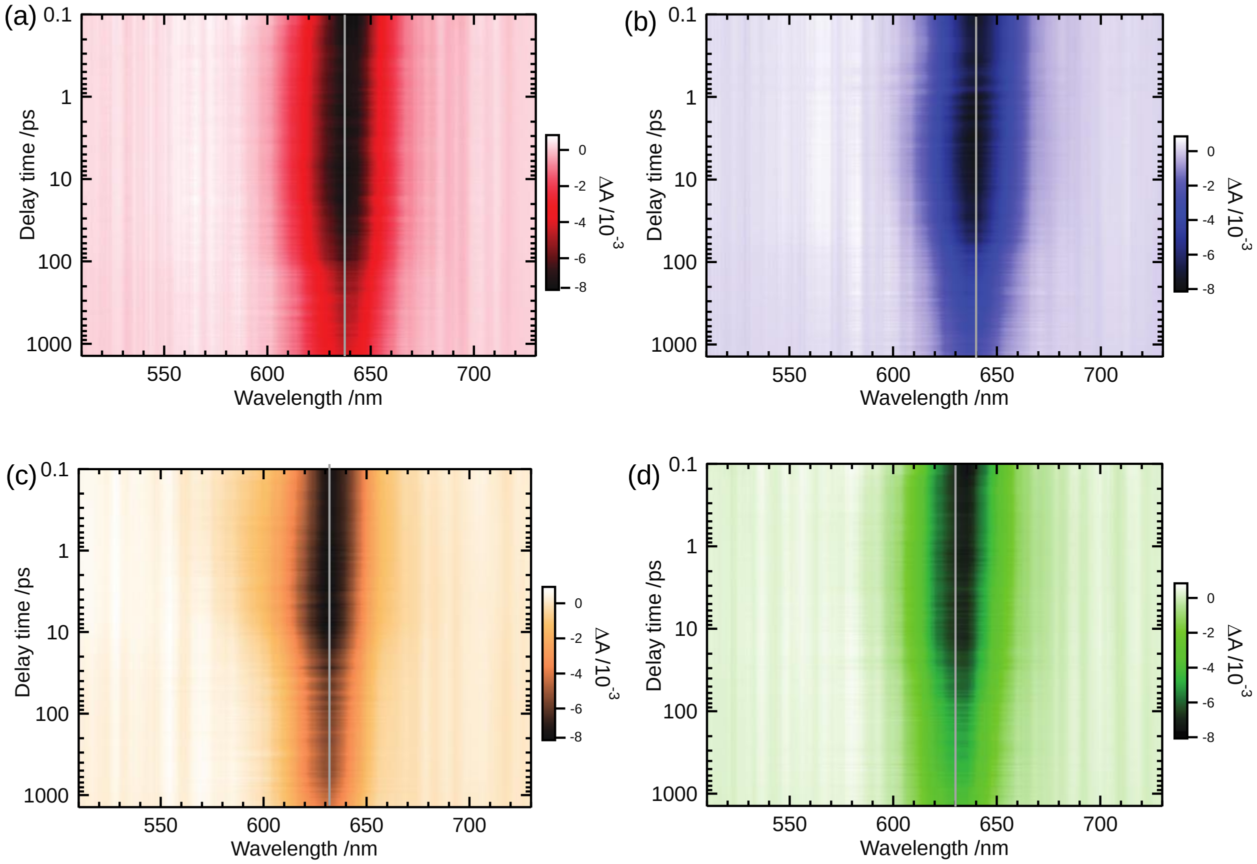

Figure S13. Femtosecond transient absorption spectrograms of ((a) Std, (b) CHA, (c) PEA and (d) TFB PeNC samples of the composition $Cs_xFA_{(1-x)}PbBr_yI_{(3-y)}$ (Std). The spectrograms were obtained using 640 nm excitation pulses

Table S1. Estimations of bandgaps and exciton binding energies obtained from UV-Vis absorption spectral band edge fitting using Elliot model equation for $Cs_xFA_{(1-x)}PbBr_yI_{(3-y)}$ (Std) and surface passivated perovskite nanocrystals with bulky organic ligands CHA, PEA, and TFB.

Sample	Bandgap<br>eV	Binding energy<br>meV
Std	1.96	6.5
CHA	1.95	1.5
PEA	1.97	3.7
TFB	1.98	5.7
